# ‘Isn't It Just a Chat?’ Allied Health Students' Experiences of Structured Clinical Supervision During Remote Placements in Australia

**DOI:** 10.1111/ajr.70173

**Published:** 2026-03-25

**Authors:** Nikki Hulse, Negin Loh, Kylie Hopkins, James Debenham, Robyn Doney

**Affiliations:** ^1^ Majarlin Kimberley Centre for Remote Health University of Notre Dame Western Australia Australia

**Keywords:** allied health personnel, health occupations, interdisciplinary placement, qualitative research, rural health, students

## Abstract

**Objective:**

This study explored allied health students' understanding and perceptions of student supervision during remote clinical placements that had adopted a newly developed structured clinical supervision model.

**Setting:**

The study was conducted in the remote Kimberley region of Western Australia.

**Participants:**

Participants were 60 occupational therapy, physiotherapy and speech pathology students who completed placements facilitated by Majarlin, a University Department of Rural Health, in the region between February and December 2021.

**Design:**

A convergent mixed‐methods design was used to study pre‐ and post‐placement surveys with Likert‐scale and open‐ended items. Quantitative data were analysed descriptively, qualitative responses were thematically analysed, and data were integrated to contextualise student perceptions across the two cross‐sectional samples.

**Results:**

Thirty‐eight students (63% participation) completed at least one survey (25 completed both surveys; 6 completed only the pre‐placement survey; and 7 completed only the post‐placement survey). Pre‐placement responses to quantitative questions commonly reflected limited or uncertain understandings of supervision; fewer than half understood their university's expectations for supervision; and only 29% had received information about the structured clinical supervision model. Post‐placement qualitative reflections frequently described supervision as a structured, reflective, and supportive process. Most students reported receiving weekly one‐to‐one and group supervision (84%), opportunities for peer‐assisted learning (88%), and supervision that incorporated reflective practice, education, and client safety (69%). Two‐thirds (66%) indicated they would recommend the model to future students.

**Conclusion:**

Students described the structured clinical supervision model as enhancing their learning, confidence and engagement with supervision during remote placements. Both qualitative and quantitative findings emphasised the value of consistent, structured supervision, including protected one‐to‐one and group supervision time. Variability in supervision delivery, particularly where students had multiple clinical educators or fieldwork supervisors, highlighted the importance of clear pre‐placement communication and consistent supervisory practices in remote contexts.

## Keypoints

- What is already known on this subject?
  ○ Rural clinical placements pose additional challenges for students and supervisors, including complex caseloads, cultural differences, geographical and professional isolation and limited availability of services. These challenges are amplified in remote locations.
  ○ Clinical supervision of allied health students during placement enhances learning, professional identity formation, reflective practice and client safety.
  ○ Limited research has explored how students understand, experience and value supervision in rural and remote settings.
- What this paper adds?
  ○ This is the first study to investigate the impact of a structured supervision model on allied health students' understanding and experience of clinical supervision during remote placements.
  ○ The findings demonstrate that structured clinical supervision strengthens student learning and wellbeing, and helps them navigate the distinctive challenges of remote placements.

## 1 Introduction

In Australia, the ratio of allied health practitioners relative to the population declines with increasing remoteness from capital cities [1]. This shortage coincides with a higher prevalence of preventable diseases in more remote regions, where allied health professionals are well placed to respond to community needs [2, 3]. Workforce shortages not only reflect existing health inequities, but also compound them, limiting timely access to care and undermining service continuity. Therefore, strengthening the rural and remote health workforce is critical. One strategy to address this need is to offer clinical placements for allied health students in these regions, exposing future practitioners to the realities and rewards of practice in diverse and often resource‐limited contexts [4].

Student placements can also generate reciprocal benefits for communities by enhancing health services, stimulating engagement and contributing new knowledge and perspectives that enhance intellectual capital [5]. In many cases, students provide services that would otherwise be unavailable. These interactions can also create the foundation for relationships between the community and the future health workforce. Evidence suggests that students who complete rural or remote placements are more likely to return to these regions for employment after graduation, supporting workforce sustainability and addressing health inequities [6, 7].

Clinical placements are a mandatory component of accredited allied health programmes in Australia, providing students with supervised, real‐world learning experiences. Placements in rural and remote areas offer students distinctive learning opportunities, characterised by a broader scope of practice, diverse service delivery, and complex social and cultural contexts compared to metropolitan settings [8]. They support personal development as students adapt to living and working in unfamiliar communities, often far from home, building core professional competencies such as clinical reasoning, communication, cultural responsiveness and resilience [9]. They also offer the opportunity to meaningfully contribute to health services in under‐resourced regions, helping students appreciate their professional roles—often working within multidisciplinary teams—and appreciate the social value of providing healthcare [10].

Although often used interchangeably, clinical supervision and student or placement supervision are conceptually distinct. Within health professions education, clinical supervision is typically defined as a reflective and developmental process that supports professional learning, facilitates reflective practice and promotes client safety and professional identity development [11, 12]. Clinical supervision may occur at either the professional or student level. In contrast, student or placement supervision is primarily educational and focuses on competency development, clinical teaching, guided practice and skill acquisition [13]. While these forms of supervision share overlapping goals and practices, they remain analytically distinct [11, 12]. In many health settings, but particularly rural and remote settings, clinical and placement supervision of students is often delivered by the same practitioner [14]. This overlap can blur boundaries for students and contribute to variability in how supervision is experienced during clinical placements. Clear articulation of supervisory roles, expectations and responsibilities is therefore critical for effective supervision and for interpreting students' experiences within complex placement models [13, 14] (Appendix).

Supervision of students is important in all settings, but it is particularly significant in rural locations where challenges can include diverse and complex caseloads, geographical and professional isolation, limited infrastructure and reduced support systems [14]. These challenges can be amplified in remote regions, where cultural considerations can add further complexity for students, making effective supervision essential for both learning and safety [15]. Supervisors also support students' emotional wellbeing and confidence, helping them navigate the challenges of remote placements and develop professional skills [16]. These attributes are transferable between practice settings [13], but are especially advantageous in remote healthcare [17].

Unlike nursing and medicine, allied health professions lack consistent national guidelines for clinical supervision, and terminology varies widely. Supervisors may be referred to as ‘educators’, ‘preceptors’, ‘facilitators’, ‘mentors’, or ‘buddies’, and these terms are often used interchangeably [18]. Such variability can create confusion for students, particularly in remote locations where large and complex caseloads make structured and consistent student supervision especially important [8, 19]. Most national guidelines and research on clinical supervision focus on supporting qualified clinicians, particularly new graduates or mid‐career clinicians [12, 20, 21]. When student supervision is explicitly considered, the emphasis is typically placed on nursing or medical contexts [13]. Currently, there are no formal national standards for guiding the supervision of allied health students in remote settings. Within the allied health literature, the perspectives of supervisors and institutions are more commonly represented than those of students [22, 23, 24, 25]. Although some studies have explored allied health student experiences in rural areas [16, 26, 27], few have examined student perspectives in remote contexts [15].

### 1.1 Setting

Majarlin Kimberley Centre for Remote Health, located in the remote Kimberley region of Western Australia, is part of Australia's national network of University Departments of Rural Health. Established in 2018, Majarlin is the Yawuru word for ‘coming back’, reflecting the goal of strengthening the local health workforce by supporting students to complete placements in the region and encouraging their return as qualified health professionals. In its first 6 years of operation, Majarlin facilitated more than 1000 student placements from over half of Australia's universities [28].

Given the variability in terminology used to describe supervision roles across clinical education contexts, the following description outlines how supervision is provided at Majarlin for the purposes of this study. Majarlin CEs (or student supervisors) facilitate community‐based, student‐led clinical placements within local communities. These placements are designed to address local health service needs while providing students with immersive exposure to the complexities and opportunities of remote practice. Students typically complete placements in pairs or small groups and are supervised either directly by Majarlin's CE or through a hybrid supervision model, in which local allied health clinicians act as onsite supervisors providing day‐to‐day fieldwork supervision under the overall responsibility of the CE/student supervisor.

### 1.2 Development of the Structured Clinical Supervision Model

Prior to the study's conception, Majarlin CEs observed inconsistent approaches between disciplines and placement sites. Feedback from students and university placement coordinators confirmed that unclear expectations and variability in supervisory practices created confusion for students.

In response, Majarlin CEs undertook a three‐stage iterative process to design a structured clinical supervision model in collaboration with local allied health clinicians. First, the lead CE (Majarlin) reviewed university course outlines. While some universities specified minimum supervision time (commonly 1 h per week), most lacked explicit or consistent expectations regarding the format, frequency and content of supervision. Second, national professional guidelines (Australian Health Practitioner Regulation Agency, Australian Physiotherapy Association, Occupational Therapy Australia and Speech Pathology Australia) were examined, which also provided limited guidance on student supervision. Third, Majarlin CEs reviewed internal supervision processes and hosted workshops to consult allied health clinicians and university representatives. A supervision model was developed to improve the transparency and consistency of expectations and processes, while remaining adaptable and pedagogically sound.

**FIGURE 1:**
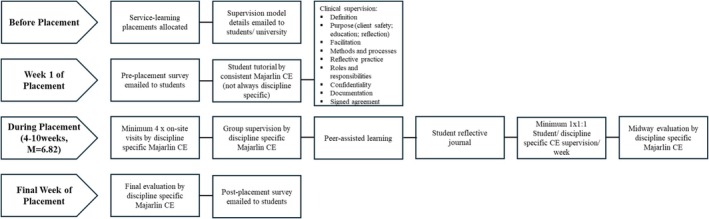
Majarlin Kimberley Centre for Remote Health Allied Health Student Structured Clinical Supervision Model.

The resulting model included the provision of information to students before placement, followed by weekly one‐to‐one and group supervision, opportunities for peer‐assisted learning, reflective journaling and at least four site visits per week by a Majarlin CE for observation and support (Figure 1). The model was incorporated into Majarlin internal governance documents and provided to prospective students and university placement coordinators.

## 2 Objective

This study explored allied health students' understanding and perceptions of supervision during remote clinical placements that adopted a newly developed structured clinical supervision model. Specifically, it examined how students defined clinical supervision and described their experiences of the structured model within the context of their placement.

## 3 Materials and Methods

### 3.1 Design

A convergent mixed‐methods design with data collected pre‐ and post‐placement was used to explore allied health students' understanding and experiences of clinical supervision within the newly developed model. Students completed online surveys before and after their clinical placement. The quantitative items assessed students' self‐reported preparedness, participation in supervisory activities and achievement of learning outcomes. Qualitative responses captured the students' definitions of clinical supervision and reflections on their experiences. As the study aimed to explore group‐level patterns in student perceptions rather than track individual change, surveys were administered anonymously, and the pre‐ and post‐placement datasets were analysed as two separate cross‐sectional samples. Data from each timepoint were analysed independently and then integrated, allowing qualitative insights to enrich and contextualise quantitative patterns to identify how students in each sample understood and experienced clinical supervision within the structured model. In this study, *structured supervision* refers to scheduled, model‐informed supervision activities with defined purposes and formats, in contrast to informal or ad hoc supervisory interactions that occur opportunistically during routine clinical work.

### 3.2 Sample

Participants were a convenience sample of allied health students who had completed clinical placements in the Kimberley region of Western Australia between February and December 2021. The eligibility criteria included students who were either fully supervised by a Majarlin Clinical Educator (CE) or placed at a site where a Majarlin CE provided all structured supervision and assessments in partnership with an onsite supervising allied health clinician who oversaw students' day‐to‐day clinical activities. The study included students from the disciplines of occupational therapy, physiotherapy and speech pathology. Students were eligible regardless of their year of study or enrolment type (undergraduate or postgraduate).

### 3.3 Survey Instrument

Pre‐ and post‐placement surveys (Table 1) were adapted from the Modified Maastricht Clinical Teaching Questionnaire, a validated tool designed to evaluate the quality of clinical teaching from the perspective of students [29]. To address the specific research objectives of this study, additional quantitative and qualitative questions were developed to capture the students' understanding and perceptions of clinical supervision. The pre‐placement survey included items on demographic characteristics, prior exposure to supervision, preparedness for placement, and students' initial definitions and expectations of clinical supervision. The post‐placement survey included equivalent items on preparedness and understanding of supervision, as well as additional items on experiences of the structured supervision model, perceived usefulness of one‐on‐one and group supervision, and alignment between supervision and learning objectives.

**TABLE 1:** Pre‐ and post‐placement survey.

**Pre‐placement survey**
Year of study^a^
Placement location^a^
Discipline^a^
Length of placement^a^
How would you define clinical supervision?^a^
*As part of my clinical fieldwork I require…* ^b^, ^c^
Consistent demonstrations as to how different tasks should be performed
Clear explanations of the important elements for the execution of a given task
Sufficient opportunities for me to observe clinical educators
A role model as the kind of health professional I wish to become
Observations of me multiple times during placement encounters
Useful feedback during or following direct observations of patient encounters
Help for me to understand which aspects I need to improve
Adjusted teaching activities to my level of experience
Sufficient opportunities to perform activities independently
Support in activities I find difficult to perform
Gradual reduced support to allow me to perform certain activities more independently
My Clinical Educator (CE) to encourage me to reflect on all aspects of my clinical practice
Encouragement to ask questions to increase my understanding
My CE to explore my strengths and weaknesses
My CE to consider how I might improve my strengths and weaknesses
Encouragement to formulate learning goals
Encouragement to pursue my learning goals
Encouragement to learn new things
My CE to create a safe learning environment
My CE to provide sufficient time to supervise me
My CE to be genuinely interested in me as a student
*Please indicate your level of agreement with the following statements…*
I have received structured clinical supervision during my previous placements^a^
My university provided me with information about Majarlin's supervision process^a^
I am aware and understand my university's expectation of clinical supervision I should receive^a^
I feel prepared to engage in the clinical supervision process during my placement^a^
Post‐placement survey
How would you define clinical supervision?^a^
*As part of my clinical fieldwork placement I received…*
Information during orientation about Majarlin's supervision process^a^, ^c^
Information that prepared me to engage in the supervision process^a^, ^c^
1:1 supervision allocated for 1 h each week^a^, ^c^
Group supervision allocated for 1 h each week^a^, ^c^
Time to engage in peer‐assisted learning to enhance my skills and knowledge^a^, ^c^
Supervision that focused on reflective practice, education and client safety^a^, ^c^
Supervision that met my learning objectives^a^, ^c^
A model of supervision that I would recommend to other allied health students^a^, ^c^

*Note:* Pre‐ and post‐placement surveys included items adapted from the Modified Maastricht Clinical Teaching Questionnaire (MCTQ) and newly developed items addressing the study objectives.

^a^ Items reported in this study.

^b^ Items adapted from the MCTQ. Identical items were included in pre‐ and post‐placement surveys with minor changes to tense.

^c^ Likert response scale (Fully Disagree, Disagree, Neutral, Agree, Fully Agree and Unable to Comment).

The surveys were piloted with five allied health students who were not part of the study cohort. Minor wording adjustments were made based on their feedback, but no changes were made to the content of the questions. The study focused on students' definitions of supervision and their perceptions of feedback within the new structured supervision model; therefore, only responses to the newly developed items were analysed and reported. Collected data included demographic variables; placement‐specific details; supervision structure, content and delivery; and the prompt (included in both the pre‐ and post‐placement surveys) ‘how would you define clinical supervision?’

### 3.4 Ethics

This study was conducted in accordance with the Australian *National Statement on Ethical Conduct in Human Research* and the principles of the Declaration of Helsinki (revised 2013). Ethical approval was granted by the University of Notre Dame Australia. Participation was voluntary and anonymous; informed consent was implied through the completion of the pre‐ and post‐placement questionnaires, which were preceded by study information outlining purpose, confidentiality and use of de‐identified data.

### 3.5 Procedure

Students were invited by the placement coordinator to complete a pre‐placement questionnaire prior to orientation at Majarlin and a post‐placement questionnaire at the end of placement. Surveys were voluntary, anonymous and confidential, and non‐participation had no impact on placement outcomes.

The pre‐placement survey was administered before any orientation activities or interactions with CEs to capture students' baseline understanding and expectations of clinical supervision. The post‐placement survey was distributed after the students' evaluations to minimise any perceived influence on their final placement assessment. Reminders were not sent to the students who chose not to complete the survey.

### 3.6 Analysis

The survey data were collated using Qualtrics (Qualtrics, Provo, UT, USA) and exported to Microsoft Excel and SPSS (version 29; IBM Corp., Armonk, NY, USA) for analysis. Descriptive statistics summarised the quantitative data, including frequencies and percentages for closed‐response items. While some questions were conceptually aligned (both included an open‐ended definition of clinical supervision), the surveys were anonymous and designed for complementary rather than paired analysis. Accordingly, the pre‐ and post‐placement surveys were not matched and were analysed as separate cross‐sectional datasets and were not used to determine changes for individual students.

Qualitative responses to open‐ended survey questions were analysed thematically using Braun and Clarke's six‐phase approach [30]. Two researchers independently coded responses using NVivo (version 14; QSR International, Melbourne, Australia) to review, discuss and refine the emerging codes. Given the nature of our data, we did not develop a hierarchical coding tree. Instead, through an iterative process, codes were grouped into overarching themes which were reviewed collaboratively to ensure conceptual clarity and coherence. Coding discrepancies were resolved through discussion until consensus was reached. Thematic saturation was informally determined once a diverse range of themes emerged. Quantitative and qualitative data were integrated with themes, allowing qualitative insights to contextualise and explain findings from quantitative data.

## 4 Results

The quantitative and qualitative findings provided an overview of student‐reported experiences and learning outcomes and offered insights into how students perceived and interpreted their supervision experience.

### 4.1 Demographics and Placement Details

Of the 60 allied health students who completed placements under Majarlin's structured supervision model during the study period, 38 (63.3%) completed either the pre‐placement (*n* = 6), post‐placement (*n* = 7) or both surveys (*n* = 25) (Table 2). Most of the respondents were female (84.2%) and from a metropolitan background (86.8%). Students were enrolled in occupational therapy (47.4%), physiotherapy (26.3%) and speech pathology (26.3%). Most were domestic students (97.4%) enrolled in undergraduate programmes (89.5%), with the majority in their final 2 years of study. The mean age was 23.2 (±2.7) years. Nearly all students (92.1%) passed their clinical placement (Table 2).

**TABLE 2:** Characteristics of students by survey completion.

Total (*n*)	Pre and post placement surveys^a^	Pre‐placement survey only^b^	Post‐placement survey only^c^	Totals (*n*; %)	
	25	6	7	38	100
Gender					
Male	6	0	0	6	15.8
Female	19	6	7	32	84.2
Age (*M*; SD)	22.8 (2.47)	23.17 (1.17)	24.86 (3.85)	23.24 (2.68)	
Rural background					
Yes	4	1	0	5	13.2
University					
Curtin	18	6	5	29	76.3
University of Notre Dame	5	0	0	5	13.2
Edith Cowan University	2	0	2	4	10.5
Discipline					
Occupational therapy	14	1	3	18	47.4
Physiotherapy	6	3	1	10	26.3
Speech pathology	5	2	3	10	26.3
Type of enrolment					
Domestic	25	6	6	37	97.4
International	0	0	1	1	2.6
Type of study					
Undergraduate	23	4	7	34	89.5
Postgraduate	2	2	0	4	10.5
Year of study					
Third	5	1	0	6	15.8
Fourth	20	5	7	32	84.2
Placement outcome					
Pass	24	6	5	35	92.1
Fail	1	0	2	3	7.9

^a^ Participants who completed both pre‐placement and post‐placement surveys.

^b^ Participants who completed only the pre‐placement survey.

^c^ Participants who completed only the post‐placement survey.

All placements were based in the Kimberley between February and December 2021. Students were grouped into small cohorts (range 2–8, mean = 4.5), with placement durations ranging from 4 to 10 (mean = 6.8) weeks. Students participated in placements within the fields of paediatrics (*n* = 22), aged care (*n* = 8), or a combination of these areas (*n* = 8). Most students were placed within a single organisation (*n* = 29), while nine students engaged in interagency placements across two service sites. Supervision structures varied across placements. Twenty four students were supervised by a single Majarlin CE throughout their placement, while 14 students were supervised by multiple CEs due to staffing changes during the study period. In some placements (*n* = 10), students also received day‐to‐day fieldwork supervision from a local allied health clinician acting as an onsite supervisor who supported students' daily clinical activities. Delivery of all components of the supervision model, however, remained the responsibility of a Majarlin CE.

### 4.2 Quantitative Survey Responses

#### 4.2.1 Pre‐Placement

**FIGURE 2:**
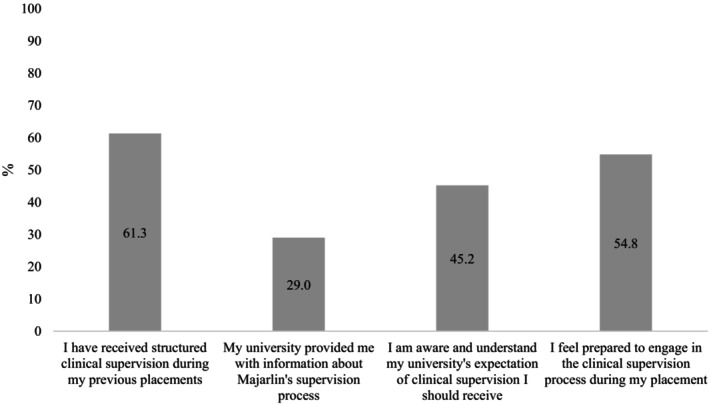
Student responses to pre‐placement survey questions (*n* = 31). Percentage of students responding ‘Agree’ or ‘Fully Agree’.

Of the 31 students who completed the pre‐placement survey, most (61.3%) reported that they had received clinical supervision during a previous (non‐Kimberley) placement. However, less than a third (29.0%) reported receiving information about Majarlin's supervision model from their university, and less than half (45.2%) understood their university's expectations for the upcoming placement (Figure 2).

#### 4.2.2 Post‐Placement

**FIGURE 3:**
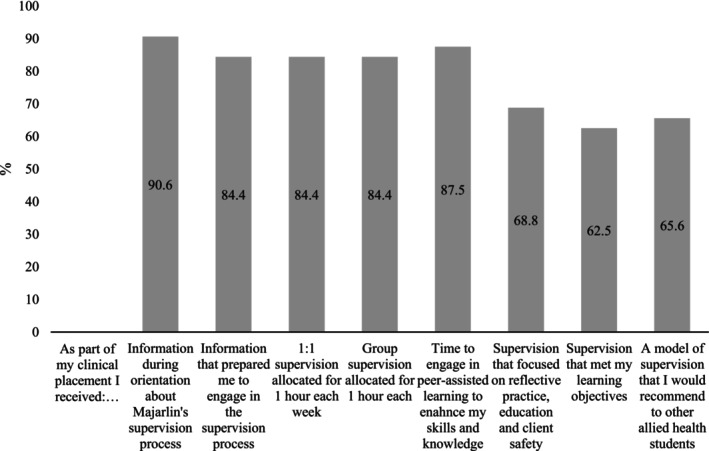
Student responses to post‐placement survey questions (*n* = 32). Percentage of students responding ‘Agree’ or ‘Fully Agree’.

Of the 32 students who completed the post‐placement survey, almost all (90.6%) reported receiving information during orientation about Majarlin's clinical supervision model, and most (84.4%) felt that this helped prepare them to engage in the supervision process (Figure 3). The majority (84.4%) also reported receiving 1 h of one‐on‐one supervision and 1 h of group supervision each week, and most (87.5%) indicated that they had opportunities for peer‐assisted learning to support skill development. Supervision included elements of reflective practice, education, and client safety for 68.8% of the students, and 62.5% felt that their specific learning objectives were fully met. Overall, two‐thirds of the students (65.6%) indicated that they would recommend the supervision model to future allied health students.

### 4.3 Qualitative Results

Thematic analysis of the open‐ended pre‐ and post‐placement survey responses identified one pre‐placement and four post‐placement core themes, offering insight into students' understanding and experiences of clinical supervision. Where relevant, qualitative findings were integrated with quantitative data to illustrate the convergence and divergence of the student responses (Table 3).

**TABLE 3:** Integrated quantitative findings and qualitative themes across pre and post‐placement surveys.

Theme	Quantitative results	Qualitative results	Example quote (anonymised participant code)^a^
Expectations and understanding of supervision	*Before placement* 29.0% had received information from their university about Majarlin's supervision process 45.2% were aware of their university's expectations regarding supervision 54.8% felt prepared to engage in the supervision process	*Before placement* Students reported understanding supervision as informal ‘checking in’	*Before placement* ‘A physio who has a chat with you on a regular basis to see how you are going’. (2501)
	*After placement* 90.6% received supervision information during orientation. 84.4% reported being prepared to engage in supervision.	*After placement* Students reported supervision as a relationship that supports growth, reflection, and professional identity. Upon reflection, many students noted that they had limited awareness of supervision prior to placement, with one describing it as ‘just a chat’. After the placement, students used terms like ‘mentorship’, ‘confidence building’ and ‘professional development’.	*After placement* ‘Support, guidance, help when needed, reflection, patient safety’. (3126)
Value of structure	84.4% received 1:1 supervision weekly 84.4% received group supervision weekly 87.5% had time for peer‐assisted learning 68.8% agreed supervision focused on reflective practice, education, and client safety	Students consistently highlighted the value of having dedicated, protected time for supervision. The structure was seen as supportive, helping them set goals, reflect on their progress, and feel emotionally supported. Many appreciated the regularity and consistency of 1:1 and group sessions.	‘I loved having supervision. It was a great way of identifying my learning needs. The one‐on‐one sessions were well planned and helpful’. (3124)
Challenges with supervision delivery	6.8% of students were supervised by more than one Clinical Educator, and 23.7% undertook multi‐site placements, both of which created variability in supervision delivery.	Several students reported inconsistencies or mixed messaged in supervision, particularly when they had more than one CE or onsite supervisors. These variations created confusion or left some students feeling unsupported.	‘It was hard to get consistent feedback across the sites. It felt like my supervisors were not communicating’. (SP002)
Supervision quality and placement outcome	62.5% agreed that supervision met learning objectives	Students often linked their perceived placement outcomes to the quality of supervision. Those who felt unsupported described limited feedback or unclear expectations.	‘I don't think I had enough supervision which resulted in me failing my placement. I did not feel supported’. (2496)
Expectations and communication	65.6% would recommend supervision model to other students	Post‐placement reflections indicated initial uncertainty about what to expect from supervision. Some felt their university had not adequately prepared them for what supervision would involve.	‘Scary first off as I did not know what to expect but I definitely valued my one‐on‐one support. I wish I was told more about this at Uni’. (3127)

^a^ Additional representative quotes are included in the Results section.

#### 4.3.1 Pre‐Placement

##### 4.3.1.1 Initial Expectations and Understanding of Supervision

Before placement, several students expressed uncertainty or held superficial definitions. For example, one student noted, ‘Not really sure… (and I'm) not even sure it is on our course content’ (3125), while another described supervision as, ‘I think it's about my supervisor giving me support and feedback’ (3126).

#### 4.3.2 Post‐Placement

In the post‐placement sample, reflections conveyed a more confident and comprehensive understanding. Students described clinical supervision as an intentional relationship centred on mentorship, feedback and personal development. One student described supervision as offering ‘guidance, support, feedback, coaching, mentoring and putting me at the centre of my learning’ (2795), while another noted its role in shaping professional identity and self‐efficacy, commenting, ‘I initially was very nervous about being in a student‐led placement but with support I now feel like an OT’ (2794).

##### 4.3.2.1 The Value of Structured and Consistent Supervision

Students consistently emphasised the value of the structured clinical supervision model, particularly the combination of individual and group‐based sessions. These supervisory activities were described as providing opportunities for guided learning, constructive feedback, and reflective discussion within a supportive environment. One student reflected, ‘Supervision can be really nerve‐racking at first because you think it's about your supervisor just telling you how to do things, but it wasn't like that at all. It was great to talk things out and be asked my opinions and thoughts. I really felt that I was invested in’ (2797). Another student described supervision as *‘me time’* (2795), underscoring the emotional and reflective benefits of having dedicated time and space to process their experiences.

##### 4.3.2.2 Inconsistencies and Challenges in Supervision Delivery

Although Majarlin clinical supervision model was generally well‐received, several students reported inconsistencies in its implementation, particularly those placed across multiple sites and thus had more than one onsite supervisor, or those supervised by more than one CE. These variations created confusion around expectations and may have led to differences in the quality and consistency of support. As one student explained, ‘It was hard to get used to supervisors' different styles… it wasn't clear what was expected’ (11248). Another similarly noted, ‘I found it really hard to switch between sites and supervisors’ (3002). However, these challenges may have reflected variability in onsite supervision, rather than the clinical supervision component delivered by Majarlin CEs.

Students also described the challenges associated with the high level of autonomy required in some community‐based, student‐led placements, particularly when onsite supervision was inconsistent or limited. One student reflected, ‘If I knew I would be spending so much time on my own I may not have chosen this placement. I struggled’ (1114), while another reported that ‘little support on‐site and feedback was not helpful as nothing was explained’ (3010).

##### 4.3.2.3 Supervision Experience Shapes Placement Outcomes

Students' perceptions of the quality of supervision were closely linked to how they experienced the placement overall. Those who did not pass or felt unsupported often attributed these outcomes to insufficient supervision or disengaged supervisors. One student shared, ‘I didn't feel like I got great supervision. My supervisor wasn't really interested in me. She didn't really teach me how to do things rather than just told me’ (3010).

Despite encountering similar challenges, other students interpreted the experience more constructively. One commented, ‘I have to be very self‐directed which has been different to other placements, but that's not necessarily a bad thing, but it should be promoted more when we choose the placement’ (1116).

##### 4.3.2.4 Navigating Expectations and Building Confidence With Communication

Post‐placement reflections indicated that many students commenced their placements with a limited understanding of clinical supervision, which in hindsight they recognised as a source of early anxiety and uncertainty. These comments were provided in post‐placement surveys as retrospective reflections on their experiences. One student noted, ‘I have never had supervision before so was a bit apprehensive and thought it would be judgemental’ (3125). Another shared, ‘I first thought that supervision would be very daunting’ (1112), while a third reflected, ‘It was hard to know what the expectations were and how to meet them’ (3002). Students described gaining clarity and confidence over the course of the placement through communication with supervisors and engagement in the structured supervision model.

## 5 Discussion

This study explored allied health students' understanding and perceptions of clinical supervision as experienced during remote clinical placements. Overall, students in the post‐placement sample reported positive experiences with the newly developed structured supervision model, emphasising the benefits of its consistency, feedback processes and reflective components. Many described increased confidence, clearer learning goals and a more developed conceptual understanding of clinical supervision in their post‐placement reflections. Conversely, some students reported that supervision under the model did not support them in meeting their learning objectives, with negative experiences more commonly described by students who experienced multiple student supervisors (CEs) across site rotations, as well as the involvement of multiple onsite supervisors.

Students' understanding of clinical supervision differed between the pre‐ and post‐placement samples. Prior to placement, many students described supervision as informal, incidental or opportunistic, whereas post‐placement reflections conveyed a more developed conception that encompassed feedback, mentorship, emotional safety and professional development. This shift suggests that participation in the structured supervision model supported students to articulate a clearer sense of purpose and value of supervision, consistent with their development as emerging professionals. These findings align with Transformative Learning Theory, which emphasises critical reflection as a mechanism for reframing perspectives [31], and with models of reflection that conceptualise reflective practice as a metacognitive process underpinning deeper learning, critical thinking and professional identity formation [32]. The results are also consistent with evidence that health professional students' reflective capacity develops over time, contributing to increased confidence and professional growth [33, 34].

Students reported that structured one‐on‐one and group‐based supervision sessions provided supportive spaces for feedback, goal setting and reflective discussions. In post‐placement reflections, these sessions were described as reducing stress, strengthening relationships with supervisors, and building confidence. Group‐based supervision and peer‐assisted learning were also perceived as reducing isolation and enabling students to support each other's learning, consistent with evidence that such approaches enhance confidence, teamwork skills and supervision efficiency [35, 36]. These findings align with previous research highlighting the value of protected, structured supervision time in supporting placement satisfaction, role clarity, learner self‐efficacy and professional identity formation [10, 37, 38].

While the structured supervision model was broadly well‐received, some students reported inconsistencies in its delivery, particularly when moving between placement sites or working with multiple CEs and onsite supervisors. These variations were perceived as creating uncertainty about expectations, reduced continuity, and contributing to confusion about the supervision process. Such experiences highlight the challenges of implementing structured supervision models in rural and remote placements, where workforce turnover, service delivery pressures and staffing variability can undermine intended consistency. These findings are consistent with the work of Martin [39] and O'Brien [40], who noted that a lack of role clarity and supervisor preparation can diminish the effectiveness of supervision. Levett‐Jones [41] similarly observed that supervisory inconsistency may increase student stress, particularly in unfamiliar or resource‐constrained environments typical of remote settings.

Some students linked their perceived placement outcomes, including perceptions of success, confidence and learning progression, to the quality of supervision they received. Those who felt well supported generally reported positive experiences, while some students who did not pass their placement or perceived themselves as unsuccessful reported inconsistent or unsatisfactory supervision. The relational quality of supervision appeared particularly influential in shaping students' confidence and perceived competence, consistent with earlier studies highlighting the importance of interpersonal support and clear role expectations [41]. A mismatch between expectations and experiences, particularly in remote contexts where students may be required to work more autonomously [42], may heighten stress and reduce engagement [43]. Preparedness is increasingly recognised as a key determinant of placement quality, underscoring the importance of clear orientation, alignment of expectations between students, educators and placement sites, and welcoming induction processes that support a sense of belonging [44, 45, 46].

### 5.1 Limitations

This study focused exclusively on students' perspectives and did not include the views of other stakeholders such as student supervisors (CEs), onsite supervisors or university placement coordinators. The findings therefore reflect students' self‐reported perceptions, which may have been influenced by individual placement experiences. Surveys were anonymous meaning that pre‐ and post‐placement responses could not be linked at the individual level; as such, the findings reflect group‐level contrasts rather than individual change over time. The sample size was modest and geographically specific to the Kimberley region, which may limit generalisability to other regions.

Qualitative data were derived from brief open‐ended survey responses. Consequently, the depth and breadth of reflection varied between participants, and responses could not be probed or clarified further. This may have limited the richness and nuance of the data compared with qualitative approaches such as interviews or focus groups, which allow for deeper exploration of participant experiences.

Despite these limitations, this study provides a foundation for future research incorporating the perspectives of student supervisors/clinical educators, onsite supervisors, and university placement coordinators, as well as examining potential service‐level and community impacts. Objective outcomes such as supervisor assessments, performance data and placement pass rates, along with longitudinal studies, may further clarify whether a structured supervision model positively influences student preparedness, confidence and workforce intentions in remote settings. Effective implementation will also depend on collaboration between universities, placement providers and supervisors, with clear communication about supervisory roles, expectations and anticipated levels of student autonomy to support informed student choices, reduce early stress and strengthen engagement with supervision.

## 6 Conclusion

Given the potential for rural and remote clinical placements to contribute to addressing allied health workforce shortages, there is a need for clearer, nationally‐consistent guidelines on student supervision that are responsive to rural and remote contexts. This study demonstrates that a structured clinical supervision model, delivered within the context of student placement supervision and incorporating reflective clinical supervision alongside competency‐based educational processes, was described by students as enhancing their learning experiences, confidence and engagement with reflective supervision during remote placements. Building on these findings, extending structured, evidence‐informed supervision models into coherent frameworks that accommodate student diversity, professional differences and the realities of remote practice may further support learning, work readiness and longer term workforce sustainability in remote regions.

## Author Contributions

**Kylie Hopkins:** writing – review and editing. **Negin Loh:** conceptualisation, methodology, writing – review and editing. **Nikki Hulse:** conceptualisation, methodology, formal analysis, writing – original draft, writing – review and editing, investigation, project administration, supervision. **James Debenham:** writing – original draft, writing – review and editing. **Robyn Doney:** formal analysis, writing – original draft, writing – review and editing, project administration.

## Disclosure

All authors are allied health professionals and clinical academics who live in the Kimberley region of Western Australia. All authors are employees of Majarlin Kimberley Centre for Remote Health (Majarlin), a University Department of Rural Health funded through the Australian Commonwealth Rural Health Multidisciplinary Training Program. Majarlin developed and implemented the supervision model described in this study. The findings from this research have been presented in part as follows: (1) 15th National Allied Health Conference (Oral Presentation) August 2023; (2) the University of Notre Dame Health Symposium (Oral Presentation) August 2023; and (3) the Occupational Therapy National Exchange (Poster Presentation) June 2024. This manuscript has not been submitted to or accepted by any other journal.

## Conflicts of Interest

The authors declare no conflicts of interest.

## Data Availability

The datasets are available from the corresponding author upon reasonable request.

## Appendix

**Table:**

Definitions	
Types of supervision	*Clinical supervision*: In this paper, clinical supervision refers to a structured, reflective process in which a supervisor and supervisee/s engage in reflective practice, explore ethical and client‐safety issues, and support professional growth and development.
	*Student supervision* (placement supervision): Supervision provided during clinical placements, focused on competency development, clinical teaching and guided practice.
Roles of supervisors	*Clinical educator*: In this study, Clinical educator (CE) refers to allied health staff who delivered the Majarlin structured student supervision model, including the provision of clinical supervision and completion of competency‐based summative assessments.
	*Onsite supervisor*: Onsite supervisors were local allied health clinicians based at placement sites who provided day‐to‐day fieldwork supervision. They did not deliver the structured clinical supervision model or complete formal student evaluations.
Majarlin	Majarlin Kimberley Centre for Remote Health
